# JOURNAL OF GERONTOLOGY SOCIAL SCIENCES: FEATURED 2021 EDITOR’S CHOICE ARTICLES

**DOI:** 10.1093/geroni/igac059.1167

**Published:** 2022-12-20

**Authors:** Jessica Kelley

## Abstract

Social science inquiry on age, aging, and the life course spans many topics and methodologies. This symposium highlights papers that were selected as Editor’s Choice articles in the Journal of Gerontology Social Sciences in 2021. These papers highlight methodological innovations, important advancements in our state of knowledge in an area, or emerging issues in the study of aging and older adults. Nguyen et al. discuss a qualitative study of the causes and consequences of financial exploitation of older adults. Newmyer et al. present a 31-country comparative study of measures of loneliness with their reliability and validity. Rurka et al. present a mixed-methods study of sibling dynamics and tensions when caring for an older parent. Dennison and Lee provide a novel method for studying intergenerational selection effects of education and older adult health. Lin and Brown demonstrate gender differences in the economic consequences of later-life divorce and potential impact of repartnering versus staying single.

